# Supplementary material for: Polyketide synthase gene clusters in the ericoid mycorrhizal fungus Oidiodendron maius: genome-wide prediction, expression profiling, and functional characterization of knockout mutants
Source: Front Plant Sci. 2026 Sep 16;17:1924430. doi: 10.3389/fpls.2026.1924430 (PMC13623903; doi:10.3389/fpls.2026.1924430)
Supplement: Supplementary file 1 [file SupplementaryFile1.zip › Supplementary Materials/Supplementary Folder 1.zip!/Oidma1_AssemblyScaffolds_Repeatmasked/knownclusterblast/cluster51/jgi.p_Oidma1_63348_mibig_hits.html]

| MIBiG Protein | Description | MIBiG Cluster | MiBiG Product | % ID | % Coverage | BLAST Score | E-value |
| --- | --- | --- | --- | --- | --- | --- | --- |
| EGD99352.1 | hypothetical\_protein | BGC0001144 | Neosartoricin\_biosynthetic\_gene\_cluster | 40 | 103.658536585 | 399 | 6.2e-111 |
| AEN83885.1 | AdaR | BGC0000156 | TAN-1612\_biosynthetic\_gene\_cluster | 39 | 116.027874564 | 378 | 1.9e-104 |
| ADI24939.1 | VrtR2 | BGC0000168 | Viridicatumtoxin\_biosynthetic\_gene\_cluster | 41 | 77.3519163763 | 315 | 1.5e-85 |
